# Quantifying cytoskeletal organization from optical microscopy data

**DOI:** 10.3389/fcell.2023.1327994

**Published:** 2024-01-03

**Authors:** Sarah Desroches, Andrew R. Harris

**Affiliations:** ^1^ Department of Mechanical and Aerospace Engineering, Carleton University, Ottawa, ON, Canada; ^2^ Ottawa-Carleton Institute for Biomedical Engineering Graduate Program, Ottawa, ON, Canada

**Keywords:** actin, cytoskeleton, microscopy, fluorescence microscopy, image analysis

## Abstract

The actin cytoskeleton plays a pivotal role in a broad range of physiological processes including directing cell shape and subcellular organization, determining cell mechanical properties, and sensing and transducing mechanical forces. The versatility of the actin cytoskeleton arises from the ability of actin filaments to assemble into higher order structures through their interaction with a vast set of regulatory proteins. Actin filaments assemble into bundles, meshes, and networks, where different combinations of these structures fulfill specific functional roles. Analyzing the organization and abundance of different actin structures from optical microscopy data provides a valuable metric for assessing cell physiological function and changes associated with disease. However, quantitative measurements of the size, abundance, orientation, and distribution of different types of actin structure remains challenging both from an experimental and image analysis perspective. In this review, we summarize image analysis methods for extracting quantitative values that can be used for characterizing the organization of actin structures and provide selected examples. We summarize the potential sample types and metric reported with different approaches as a guide for selecting an image analysis strategy.

## Introduction

Actin filaments (f-actin) are slender polymers (7–9 nm width, up to microns long) assembled from globular subunits (g-actin). In cells, actin filaments dynamically assemble and disassemble through the interaction of g-actin and f-actin with a range of regulatory proteins that include actin nucleating, severing and capping proteins. Other actin regulatory proteins assemble actin filaments into higher order structures by facilitating interactions between actin filaments, for example, through branching from the side of a filament, bundling or crosslinking filaments together. Different types of actin structure fulfill specific functional roles. For example, stress fibers are contractile actin bundles composed of actin filaments with alternating polarities (Naumanen et al., 2008). Repeating units of non-muscle myosin II (NMMII) exist within these bundles and interact with actin filaments, ultimately producing contractile forces within the cell (Elosegui-Artola et al., 2014; Lehtimäki et al., 2021). Thus, stress fibers are responsible for cellular processes such as migration, adhesion, morphogenesis, and mechanosensing of external forces via protein complexes at focal adhesions (Lehtimäki et al., 2021). In contrast, lamellipodia are sheet-like membrane protrusions located at the leading edge of motile cells (Verkhovsky et al., 2003; Mattila and Lappalainen, 2008). These protrusions are composed mainly of branched actin networks responsible for protrusion and retraction of the cell membrane, which allow cells to explore their local environment during migration (Zimmermann and Falcke, 2014).

Quantification of the abundance and organization of actin structures can provide a significant amount of information about the physiological state of a cell. For example, stress fiber density is believed to be proportional to a cell’s ability to spread (Elosegui-Artola et al., 2014), and quantifying the abundance and orientation of stress fibers has been used to characterize cellular responses to the mechanical environment. In addition to characterizing the physiological state of the cell, quantification of actin structures can also provide insights into the mechanisms of disease. For example, filopodia are involved in driving cellular migration. An increase in the number and length of these structures is believed to be associated with an increased risk of metastasis (Nilufar et al., 2013). While the ability to quantify actin is beneficial for understanding both physiological and pathological states, it remains a challenging task and an active area of research.

Since cellular actin structures consist of tens to hundreds of filaments, measuring their size and abundance is possible with optical microscopy and within the resolution of conventional fluorescence microscopes. Fluorescence microscopy techniques including widefield, confocal and more recently super-resolution microscopy are widely used for obtaining images of cytoskeletal organization. A range of probes have been developed for fluorescently labelling actin structures. The gold standard fluorescence probe for imaging actin filaments in fixed cells is fluorescently conjugated phalloidin. Phalloidin specifically binds to F-actin with high affinity allowing images with high signal to background to be obtained (Wulf et al., 1979; Adams and Pringle, 1991). Phalloidin staining is generally regarded to provide faithful labelling of different types of actin structures and provides similar images to cells stained with actin antibodies. While phalloidin staining is used for imaging actin organization in fixed cells, a range of different actin probes have been developed for visualizing the actin cytoskeleton in live cells (Melak et al., 2017). Live cell probes for visualizing actin include GFP fusions to G-actin (Westphal et al., 1997; Doggett and Breslin, 2011), fusions to actin-binding proteins or actin-binding peptides (Burkel et al., 2007; Riedl et al., 2008; Lopata et al., 2018; Harris et al., 2020), or live dyes (Lukinavičius et al., 2014) and the advantages and disadvantages of each of these techniques has been discussed previously (Melak et al., 2017).

**FIGURE 1:**
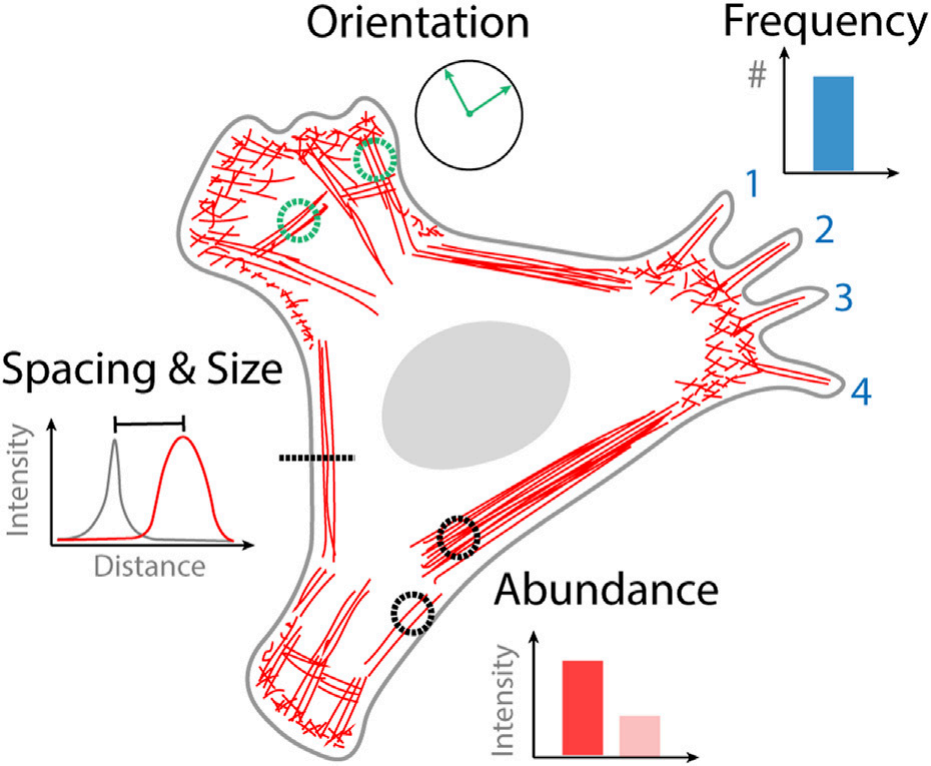
Quantitative information obtained from images of cytoskeletal organization. Frequency of actin structure occurrence that can be counted, for example, the number of filopodia. Orientation of cytoskeletal structures, that might become aligned in the direction of external or internal stimuli such as stress fibers. The spacing and size of different actin structures for example, the proximity of the actin cortex to the plasma membrane. The abundance of actin in a different structure or the density of actin binding protein bound to that structure.

Quantifying images obtained from optical microscopy can either be done manually, semi-automated or automatically. Manual analysis of an image involves counting specific structures that are identified by the user or making measurements from regions of interest that are drawn onto an image. Automated analysis requires no user intervention in the analysis step where image data can simply be streamed to an algorithm. Semi-automated analysis involves an initial user step followed by automated analysis, for example, initially selecting a cell to be analyzed which is then subsequently analyzed by an algorithm. The type of data that can be obtained from quantifying cytoskeletal organization varies based on the goals of the study but can generally be broken down into four categories; the frequency of occurrence of a particular structure, the relative orientation of structures, the spacing and size of different structures, and the abundance of actin or binding proteins contained within a structure (Figure 1). In the following sections we describe selected examples of analysis strategies based on the type of actin structure that they have been designed to quantify.

### Stress fibers and focal adhesions

Three subclasses of stress fibers exist and differ based on their association with focal adhesions. Ventral stress fibers are the most predominant subclass and are attached to focal adhesions on both ends (Lehtimäki et al., 2021). Ventral stress fibers are typically responsible for changes in cellular shape, adhesion, and overall cellular contractions (Naumanen et al., 2008). Dorsal stress fibers are attached to focal adhesions at one end while the other end of the actin bundle extends towards the dorsal side of the cell (Naumanen et al., 2008). This subclass is not necessarily contractile itself, but rather interacts with other subclasses to propagate contractile forces (Naumanen et al., 2008). Transverse arcs are the third stress fiber subclass and exist along cellular edges near lamellipodial actin networks (Lehtimäki et al., 2021). These stress fibers are thin contractile actomyosin bundles that do not directly interact with focal adhesions (Naumanen et al., 2008; Lehtimäki et al., 2021). Contractile forces generated across transverse arcs are transmitted to dorsal stress fibers, and then further propagated to focal adhesions (Lehtimäki et al., 2021). The ability to quantify stress fibers is critical as these structures play key roles in both detecting and transmitting mechanical forces to the extracellular matrix via focal adhesions, therefore dictating cellular behaviors (Elosegui-Artola et al., 2014). Their phenotypes and architecture are also key indicators of current cellular processes, both in normal and pathological states (Zhang et al., 2017).

Stress fiber extractor (SFEX) is an open-source image processing software developed by Zhang et al. (2017) which reconstructs and subsequently quantifies actin stress fibers. Firstly, cytoskeletal structures in microscopy images are enhanced to facilitate binarization. Skeletonized images are then generated, containing linear stress fiber fragments. The second part of this algorithm works to reconstruct traces of stress fibers in an iterative manner by searching for fragment pairs. This method results in a reconstructed image where quantitative values, such as fiber width, length, orientation, and shape can then be obtained. Stress fiber width is an important indicator of cellular mechanical properties, as width is believed to be correlated with cell contractility and additional actin regulatory pathways.

FSegment is a stress fiber quantification tool developed by Rogge et al. (2017) capable of analyzing changes in stress fibers over time. This algorithm specifically focuses on extracting metrics such as stress fiber length, width, orientation, and intensity distribution. Several pre-processing steps are first completed to segment the stress fibers, followed by subtraction of the non-fiber region in the image resulting in a fiber mask where the above parameters are quantified. Similar to the previously described stress fiber quantification algorithm, the output parameters provided by this algorithm are useful in describing various cellular processes. For example, stress-fibers are often thicker under instances of actin polymerization or when contractility is being upregulated (Zhang et al., 2017). Alternatively, these structures are thinner when the cell is in a relaxed state (Zhang et al., 2017).

SFALab is a recent image analysis algorithm developed by Mostert et al. (2023) which segments focal adhesions and identifies ventral stress fibers. This algorithm first generates a cell mask to determine focal adhesion density per cell and confirm that only focal adhesions within the same cell are being analysed. Shape fitting is then used to identify focal adhesion structures, which are then analyzed for morphological features such as area and aspect ratio. The original gray scale image is enhanced and combined with the segmented focal adhesions. Curve fitting is performed on the combined image between focal adhesion pairs to identify ventral stress fibers where a polynomial with the highest mean intensity to the input image is used. Parameters such as number of ventral stress fibers and ventral stress fibers per focal adhesion are provided at this stage. This technique expands on a similar algorithm by Elosegui-Artola et al. (2014), and is useful as it specifically focuses on ventral stress fibers which play a key role in transmitting forces to and from the extracellular matrix due to their interaction with focal adhesions. Focal adhesion density within a cell is believed to be related to the degree of tension a cell is supporting, as more focal adhesions provide more attachment points for stress fibers (Elosegui-Artola et al., 2014).

### Cortical actin

The actin cortex is a thin (∼150–200 nm thick) meshwork of filaments located beneath the plasma membrane (Chugh and Paluch, 2018). Within the actin cortex, filaments are densely crosslinked together through the activity of actin binding proteins including filamins, actinin, and myosin (Biro et al., 2013; Vadnjal et al., 2022). A major functional role of the actin cortex involves determining cell shape and mechanics as this structure is critically important for the generation of mechanical forces that drive both cell migration and division (Eghiaian et al., 2015; Chugh et al., 2017; Kelkar et al., 2020). Characterizing the organization of the actin cortex is therefore crucial for understanding the fundamental mechanisms of cellular force generation shape change. To date, most of the work has focused on quantifying two characteristics of the actin cortex, the pore size of the meshwork, and the thickness of the meshwork beneath the plasma membrane.

The cortical actin network is densely packed with a pore size that is typically below the resolution limit of standard fluorescence microscopy techniques and has instead been measured from electron microscopy images (Bovellan et al., 2014), or super-resolution imaging (Xu et al., 2013). The mesh size of the network has been characterized by scanning electron microscopy to be ∼30 nm in control conditions. Perturbations of actin filament assembly and nucleation lead to increases in mesh size and concurrently a reduction in the density of actin filaments. For example, when the activity Arp2/3 complex and formin mDia1 are perturbed using shRNA or pharmacological inhibitors the mesh size of the cortex increases from ∼30 nm to ∼100 nm. In contrast to the meshwork pore size, fluorescence microscopy has been successfully used to measure the thickness of the actin cortex. Changes in the distribution and organization of actin filaments and actin binding proteins within the cortex lead to gradients in mechanical tension within the cell cortex that drive cellular shape changes (Truong Quang et al., 2021). Measuring cortical thickness and the localization of different actin regulatory proteins throughout the cortex is fundamental for understanding this process. Seminal work by Clark et al. (2013) measured the thickness of cortical actin in HeLa cells to be on average ∼190 nm. Thickness measurements were obtained by measuring the position of the plasma membrane and cortical actin which were labelled with mCherry-CAAX and GFP-actin respectively. Position measurements were obtained by determining the peaks of the fluorescence intensity along linescans from the two-color channels which can then be used to determine the thickness of the actin cortex beneath the membrane. This technique leverages the rounded shape of mitotic cells and the ability to fit the fluorescence intensity profile of the linescan to obtain thickness measurements that are below the diffraction limit. However, this technique uses the assumption of uniform actin distribution through the cortex and requires additional calibration steps to correct for sources of chromatic aberration and differences in background fluorescence between the color channels. Clausen et al. (2017) advanced on this technique, using STED microscopy to provide higher resolution images that could then be used to determine the spacing of cortical actin from the membrane. The authors found an asymmetric distribution of actin density suggesting a maximum spacing of 20 nm of cortical actin from the membrane, with below 10 nm in some regions. In addition to measurements of thickness and proximity to the plasma membrane, STORM imaging has been used in a similar approach to measure the distribution of actin regulatory proteins within the actin cortex. Measuring differences in the intensity profile of fluorophores targeted to different proteins or the plasma membrane presents a valuable technique for precisely measuring distances, that has been applied not only to the actin cytoskeleton but also for characterizing membrane protein height (Son et al., 2020).

### Lamellipodia, filopodia, and podosomes

Lamellipodia are sheet-like membrane protrusions located at the leading edge of motile cells composed of branched actin networks (Verkhovsky et al., 2003; Mattila and Lappalainen, 2008). Actin orientation within lamellipodial structures is fundamentally linked to the direction in which a cell is moving (Verkhovsky et al., 2003; Zimmermann and Falcke, 2014). An image processing algorithm developed by Verkhovsky et al. (2003) quantifies actin orientation in lamellipodial protrusions at both an ultrastructural and cellular level. This algorithm utilizes a combination of electron-microscopy images, phase-contrast light microscopy images, and fluorescent images stained with phalloidin. A single cell is captured using at least two of the three techniques and the images are aligned with one another. Images are first pre-processed for noise removal and subsequently subjected to one of two edge detection methods, either a glowing edge filter or Canny filter. All resultant images are then superimposed to produce a final image accounting for edges at all orientations. Both edge detection methods ensure uniform sensitivity to edges of features at varying orientations, as some methods preferentially detect edges at a vertical or diagonal orientation rather than horizontal, which becomes problematic when quantifying structures such as lamellipodia. Following these pre-processing steps, a Radon transform is applied using a rotating square mask to isolate the specific region of interest (ROI) being analyzed. This function produces a strong signal if the linear features in the ROI are orientated in the direction of the current projection, or in other words, the actin filaments within the lamellipodia being analyzed.

Embedded within lamellipodia are filopodia, or protrusive structures composed of parallel bundles of actin filaments (Mattila and Lappalainen, 2008). These thin finger-like protrusions are responsible for probing their microenvironment to assist in sensing the surrounding environmental conditions. While filopodial size often depends on cell type, these structures typically do not exceed 10 μm in length (Mattila and Lappalainen, 2008). Filopodia numbers can indicate the current migratory state of a cell, which is important in pathological scenarios such as metastatic cancers (Nilufar et al., 2013).

FiloDetect is an automated tool for quantifying filopodia in fluorescent images developed by Nilufar et al. (2013). This algorithm detects, counts, and measures filopodia using intensity-based thresholding and various morphological operations. Cell bodies are first segmented using an intensity thresholding technique which ultimately eliminates any background pixels detected as part of the foreground. Once segmentation has been completed, morphological operations are performed where any fragments removed from the main cell body are defined as candidate filopodia. These structures are then defined as filopodia if they pass a specific size threshold and fit to an ellipse. Filopodia length is then calculated by thinning each individual structure to a single pixel width, and then counting the remaining number of pixels. A filopodia count can also be determined at this stage of the algorithm. FiloQuant is another filopodia detection algorithm developed by Jacquemet et al. (2017) capable of detecting filopodial protrusions in both fixed and live cell microscopy data. This ImageJ plugin provides step-by-step user validation to ensure appropriate segmentation of these small structures. Intensity based thresholding is first applied to the image to define cell edges and create a mask while removing any filopodial-like structures surrounding the cell. In parallel, the original image is enhanced. These two images are then superimposed to produce a resultant image containing only structures surrounding the originally defined cell mask, which are defined as filopodial protrusions. Skeletonization is performed on the resultant image to determine filopodia length. Filopodia density is also determined at this stage by calculating a ratio of filopodia count to cell edge length, which is a useful metric in the context of cancer metastasis.

Podosomes are actin-rich structures that play a role in cell migration and invasion (Linder et al., 2023). These structures release proteolytic enzymes which degrade the extracellular matrix (Linder et al., 2023). Podosomes have a dense F-actin core with a diameter of approximately 350 nm and are surrounded by a 250 nm wide ring composed of both integrins and integrin-associated proteins (Linder et al., 2023). In physiological conditions, podosomes are critical for cell invasion across tissue boundaries for effective immune surveillance (Linder et al., 2023). Podosomes are often difficult to distinguish from other actin rich structures due to their smaller size and the heterogeneity of podosome core intensities (Meddens et al., 2013).

A quantitative image analysis algorithm developed by Meddens et al. (2013) separates podosome cores from other F-actin structures in phalloidin-stained images based on intensity, shape, and size. Images are first pre-processed by Gaussian smoothing, high pass filtering, and unsharp masking resulting in enhanced edges. A local threshold is then applied to achieve foreground separation, and analysis of roundness and area is also performed to filter out any objects not resembling podosome cores. This resultant image is then combined with a Gaussian smoothed version of the original image to further filter out any objects appearing smaller than podosomes. Foreground refinement is achieved by additional thresholding. A final podosome core mask is produced by performing a watershed segmentation on the distance transform of the binary image to isolate any podosome cores that may be touching or connected with one another. At this stage, podosome size and shape are calculated from the image mask while intensity is calculated by analyzing pixel intensity values within the ring surrounding each podosome structure.

## Discussion

Quantitative characterization of the organization of the actin cytoskeleton into different structures is critical for our understanding of both normal physiology and disease. A broad set of techniques have been developed to accomplish this challenging task and we have summarized a subset of these in relation to different structures that they are designed to analyze (Table 1). New algorithms are continuously being developed, but all the approaches face similar challenges in sample preparation, imaging acquisition, and dealing with cell heterogeneity, which we discuss below.

**TABLE 1:** Summary of analysis strategies.

Structure	Example of sample type	Metric	Example of output	Potential application	References
Multiple	2D analysis of human mesenchymal stromal cells	Intensity	F-actin distribution across a cell (or across the nucleus)	Pharmacological perturbations	Zonderland et al. (2019)
	2D analysis of immortalized retinal pigmented epithelium cells	Intensity, orientation, spacing, and size	Quantifies mesh hole size, hole circularity, distance between junctions, filament density and length	cell-cell junctions	Flormann et al. (2021)
	2D/3D analysis of HeLa cells	Intensity, orientation, and size	Identifies centerlines of biopolymer networks and network junctions	Temporal evolution of biopolymers	Xu et al. (2015)
	2D analysis of a KR158 astrocytoma cell line	Intensity and orientation	Peripheral actin bundles, stress fibers, internal punctate, or protrusive actin	Pharmacological perturbations	Lockett et al. (2014)
	2D analysis of MG-63 osteoblasts	Orientation and size	Filament length and orientation	Adhesion to Biomaterials	Matschegewski et al. (2012)
	2D analysis of normal human dermal fibroblasts	Intensity and spacing	Actin abundance measurements	Response to substrate stiffness	Alhussein et al. (2016)
	2D analysis of human umbilical vein endothelial cells	Intensity and orientation	Orientation and density of actin fibers	Response to mechanical stretch	Yoshigi et al. (2003)
	2D analysis of onion epidermal cells	Intensity and orientation	Orientation and anisotropy of fibrillar structures	Analysis of orientation and anisotropy	Boudaoud et al. (2014)
	2D analysis of cardiac fibroblasts	Intensity and spacing	Measures uniformity of actin organization	Response to mechanical stretch	Fuseler et al. (2007)
	2D analysis of osteoblasts	Orientation and size	Filament orientation, filament position, and filament length	Response to fluid shear stress	Alioscha-Perez et al. (2016)
	2D analysis of NIH/3T3 cells	Intensity and orientation	Distribution of actin filaments and average quantity of actin per cell	Pharmacological perturbations	Liu et al. (2018)
	2D analysis of osteoblasts	Frequency and size	Total filament length, maximum filament length, and mean filament length	Adhesion to Biomaterials	Gruening et al. (2021)
	2D analysis of NIH/3T3 cells	Intensity	F-actin intensities per cell and relates these values to mechanical measurements	Pharmacological perturbations	Liu et al. (2020)
	2D analysis of mesenchymal stem cells	Frequency/abundance and spacing	Detects changes in F-actin structures (e.g., bundle or cross-linked)	Pharmacological perturbations	Revittser et al. (2021)
	2D analysis of melanoma cells	Abundance and spacing	Quantifies elongation and density of actin patches	Cancer progression	Sheykhi et al. (2022)
	2D analysis of human osteosarcoma	Intensity	Quantifies changes in actin filament organization	Response to cancer therapies	Vindin et al. (2014)
Stress Fibers and Focal Adhesions	2D analysis of human osteosarcoma cells	Orientation and size	Stress fiber width, length, orientation, and shape	Quantification of stress fibers in cells plated on fibronectin micropatterns	Zhang et al. (2017)
	2D analysis of A549 cells, H460 cells, and H1299 cells	Orientation, spacing, and size	Angular distribution of stress fibers	Cancer progression	Basu et al. (2022)
	2D analysis of murine podocyte cells	Intensity, orientation, and size	Stress fiber length, width, orientation	Pharmacological perturbations	Rogge et al. (2017)
	2D analysis of JC-53 cells	Intensity, abundance, and orientation	Uses coherency to analyze actin density per cell area, relative coherency per cell area, and mean coherency per image	Quantification of the actin cytoskeleton’s role upon HIV-1-entry	Weichsel et al. (2010)
	2D/3D analysis of HeLa cells	Intensity, frequency, abundance, size	Focal adhesion area, circularity, mean intensity, density per cell and actin stress fiber count	Pharmacological perturbations	Elosegui-Artola et al. (2014)
	2D analysis of human epicardial-derived cardiac fibroblasts	Intensity, orientation, frequency, spacing, and size	Quantifies number of ventral stress fibers	Pharmacological perturbations	Mostert et al. (2023)
	2D analysis of Swiss 3T3 fibroblasts	Intensity, orientation, frequency, abundance, and size	Number of fibers, length of fibers, density of fibers, and fiber polarity	Pharmacological perturbations	Lichtenstein et al. (2003)
Cortical Actin Network	2D analysis of bovine aortic endothelial cells	Frequency, spacing, and size	Quantifies the number of holes, mean area of holes, and overall surface coverage of holes	Pharmacological perturbations	Kronlage et al. (2015)
	3D analysis of AF549 cells	Intensity and size	Quantifies the area and perimeter of spaces between actin filaments in a network	Pharmacological perturbations	Garlick et al. (2022)
	3D analysis of Jurkat T-cells stably expressing either LifeAct-Citrine or LifeAct-SNAP.	Intensity and spacing	Quantifies spacing of cortical actin from the membrane subcellular regions	Analysis of cortical actin membrane dynamics and spacing	Clausen et al. (2017)
	2D analysis of HeLa cells	Intensity and spacing	Quantifies actin cortex thickness	Pharmacological perturbations	Clark et al. (2013)
	3D analysis of actin purified from rabbit skeletal muscle	Spacing/size	The mean mesh size is determined for actin network bundles using z-stacks	Pharmacological perturbations	Cavanna and Alvarado (2021)
Lamellipodia, Filopodia, and Podosomes	2D analysis of B16F1 mouse melanoma cells, and BT549 human breast cancer cells	Frequency and size	Length of filopodial structures	Cancer progression	Nilufar et al. (2013)
	2D analysis of astrocytes	Frequency and size	Number and length of filopodia in astrocytes	Pharmacological perturbations	Aumann et al. (2017)
	2D analysis of multiple cell types	Intensity, frequency, and size	Filopodia length, straightness, tip movement, base movement, dynamics	Filopodial structures in growth cones	Urbančič et al. (2017)
	2D analysis of immortalized normal breast epithelial cells (MCF10A)	Intensity, abundance, and size	Quantifies filopodial protrusion dynamics, density, and length	Cancer progression	Jacquemet et al. (2017)
	2D analysis of black tetra keratocytes	Orientation	Quantifies the orientation of actin filaments in lamellipodial protrusions	Combination of fluorescence, phase contrast, and electron microscopy	Verkhovsky et al. (2003)
	2D analysis of human dendritic cells generated from monocytes	Intensity, frequency, and size	Podosome core intensity, size, and shape	Pharmacological perturbations	Meddens et al. (2013)

Significant consideration needs to be taken when preparing samples and labelling actin structures. For fixed cells, actin structures can be preserved during sample preparation using a specific combination of fixatives and buffers (Abe and Davies, 1995). For imaging, two major considerations need to be made when choosing a probe for F-actin. Firstly, does the use of the probe impact the organization of actin filaments into different structures? For example, GFP-fusions to actin can perturb actin filament assembly, and dyes that are based on small molecules can have stabilizing effects on F-actin impacting actin filament disassembly. Secondly, does the reporting probe faithfully label all actin structures? For example, fluorescent fusions to actin binding proteins and their minimal actin binding domains have been shown to only label a subset of actin structures. This effect has been attributed to competitive interactions with endogenous proteins (Gunning et al., 2008), overall actin binding affinity and dynamics (Harris et al., 2019) and specificity to different populations of actin filaments (Harris et al., 2020). The probe used for reporting the formation of different actin structures must therefore be carefully selected and can be compared against fluorescently conjugated phalloidin in fixed cells to determine labelling efficiency.

While light microscopy techniques have served as a valuable tool for many years, the ability to image detailed subcellular structures and organelles is still often hindered by the diffraction limitation (Jing et al., 2021). As a general principle, imaging of actin structures requires high resolution imaging obtained with a high numerical aperture objective. This enables an image with a resolution that provides sufficient sampling of the structure that is being analyzed (for example, filopodia are 0.2–0.4 µm in diameter, requiring an image with a pixel size of <0.2 µm, at the limit of diffraction limited optical microscopy). Because actin is one of the most highly abundant proteins, sectioning techniques such as confocal microscopy can reduce background fluorescence in the image allowing for improved signal to noise and ultimately a more successful analysis. Super-resolution microscopy (SRM) has become a more commonly used approach for imaging subcellular structures such as actin filaments. SRM can detect smaller features, providing more information about actin structures than what has previously been available.

Cells vary significantly in both shape and size, making the initial segmentation of cell boundaries and quantification of metrics that characterize actin structures between cells challenging. For this reason, semi-automated analysis of cell images acts as a useful compromise between automated and manual image processing algorithms. Semi-automated analysis allows for the user to define certain parameters to cope with heterogeneity of cell images, ensuring algorithm specificity to the experiment and imaging conditions while limiting the amount of time spent and potential human error created while processing image datasets. In addition, normalization processes are often implemented into image processing algorithms as an effort to quantify structures independent of overall cell shape and size. In recent years, a number of machine learning algorithms have been released using various methods to both quantify and/or classify cell shape and morphological features (Kan, 2017; Li et al., 2021; Liu et al., 2021; Phillip et al., 2021). One possibility could be to combine automated machine learning approaches for identification of cell shape and area as a pre-processing step when quantifying cytoskeletal organization. Indeed, user-friendly software packages to accomplish these tasks such as CellProfiler (Jones et al., 2008; Dao et al., 2016) and Ilastik (Berg et al., 2019) are becoming increasingly common.

## Conclusion

The actin cytoskeleton is critical for many physiological processes, including but not limited to driving cell motility, determining cellular shape, and sensing and transmitting mechanical forces. Actin filaments organize into higher order structures to achieve these functions, including stress fibers, cortical actin networks, lamellipodia, filopodia, and podosomes. The ability to quantify these actin structures in terms of intensity, orientation, frequency, abundance, spacing and size, is therefore essential to understanding the current state of cells in both physiological and pathological conditions. While a significant amount of information can be obtained by quantifying the actin cytoskeleton, many challenges exist, including dealing with heterogeneity of cell shape and size, and difficulties distinguishing actin-rich structures from one another. The field of actin quantification will continue to evolve with the increased use of machine learning and SRM, which will ultimately improve our ability to quantify and understand this cytoskeletal structure.
